# Effects of V and Co Element Addition on Microstructures and the Mechanical Properties of In Situ Biphasic Hybrid (TiC_x_N_y_–TiB_2_)/Ni Cermets

**DOI:** 10.3390/ma11091750

**Published:** 2018-09-17

**Authors:** Feng Qiu, Xiangzheng Duan, Xiujuan Li, Hongyu Yang, Yawei Wang

**Affiliations:** 1 Key Laboratory of Bionic Engineering, Ministry of Education, Jilin University, Changchun 130025, Jilin, China; qiufeng@jlu.edu.cn (F.Q.); duanxz17@mails.jlu.edu.cn (X.D.); 2 Key Laboratory of Automobile Materials, Ministry of Education, Jilin University, Changchun 130025, Jilin, China; ywwang@mails.jlu.edu.cn; 3 Department of Materials Science and Engineering, Jilin University, Changchun 130025, Jilin, China; 4 Qingdao Automotive Research Institute of Jilin University, Qingdao 266000, Shandong, China; 5 School of Materials Science and Engineering, Jiangsu University of Science and Technology, Zhenjiang 212003, Jiangsu, China; yanghy@just.edu.cn

**Keywords:** combustion, in situ, cermets, compression property

## Abstract

In situ micro-(TiC_x_N_y_–TiB_2_)/Ni cermets with different Co and V content (2,5 and 8 wt.%) were successfully fabricated by combustion synthesis and hot press consolidation in Ni–(V/Co)–Ti–B_4_C–BN systems. The results indicate that as Co content increased from 0 to 8 wt.%, the average sizes of the ceramic particles decreased, when the content of V increased from 0 to 8 wt.%, the size of the ceramic particles first decreased and then increased, and when the V content is 5%, the ceramic particle size is the smallest. The Co element did not participate in the SHS reaction and was a diluent; therefore, when the Co element was added, the combustion temperature continued to decrease. When the V content was no more than 5 wt.%, as the V content increased, the maximum combustion temperature decreased. When the content of V was less than 5 wt.%, the concentration of V was not sufficient to greatly promote the generation of VN. Therefore, V absorbed a large amount of heat during the reaction, resulting in a continuous decrease in the reaction temperature of the reaction system during the reaction. When the content of the added V continued to increase to 8 wt.%, V participated in the reaction, which was exothermic. The results indicate that as Co content increased from 0 to 8 wt.%, the average sizes of the ceramic particles decreased, and the cermets with 5 wt.% Co possessed the best comprehensive properties: the highest hardness (1967 Hv), superior compression strength (3.25 GPa) and higher fracture strain (3.3%). Correspondingly, when the V content was 8 wt.%, the ultimate compressive strength and hardness of the cermets reached 1823 Hv and 3.11 GPa, respectively, 262 Hv and 0.17 GPa higher than those of the unalloyed cermets, respectively. Furthermore, the effects of Co and V on strengthening mechanisms were analyzed.

## 1. Introduction

In recent years, with the development of the aerospace, automotive, exploration and mining industries, higher requirements have been placed on the performance of materials. In the aviation field, the thrust-to-weight ratio is an engine’s core parameter and directly affects an aircraft’s various indicators [1,2]. As the thrust and efficiency of the engine increase, the engine’s turbine inlet increases, as do the requirements for high-temperature and corrosion-resistant materials [3,4]. Among the tool materials in the exploration industry, the current high-speed and high-efficiency cutting tools are mainly superhard tools (including ceramics, diamond and cubic boron nitride (CBN)) and cermet tools [5,6,7,8]. The materials used to prepare such tools require high hardness, high wear resistance, sufficient strength and toughness, high heat resistance and good processability and economy. Ceramic matrix cermets have a high melting point, stiffness and hardness and are resistant to creep and fatigue [9,10,11,12]. This not only helps to overcome the high density and low temperature resistance of metal materials, but also surmounts shortcomings such as the brittleness of structural ceramics [13,14,15]. Therefore, ceramic matrix cermets have become the research focus of relevant scholars in recent years. TiB_2_ and TiC_x_N_y_ ceramics are widely used in the preparation of electrodes and cutting tools because of their high hardness, high modulus, high electrical conductivity, high melting point, low density and excellent chemical stability [16,17,18]. In addition, the compression performance and wear resistance of two-phase ceramic particle reinforced cermets are higher for single-phase ceramic particle reinforced composites [19,20]. Ni has excellent plasticity, ductility and impact resistance and high temperature and corrosion resistance, and the wettability between Ni and ceramic phase is good [21]. Therefore, Ni as a binder with biphasic ceramics as the matrix of cermets has better strength, hardness, wear resistance, oxidation resistance and adhesion resistance [22,23,24]. At present, many studies and reports have considered Ni-based composites with TiC_x_, TiB_2_ and TiC_x_N_y_ as single-phase reinforcements [25]. Furthermore, the addition of alloying elements can change the wettability of ceramic particles and matrices, which can affect the density and microstructure and change the hardness, strength and toughness of the cermets [26,27,28,29,30]. The current research on the addition of alloying elements concentrates mostly on molybdenum and tungsten; few studies have considered the addition of Co and V in examining the microstructure and properties of cermets [31]. Co and V are metal elements with a high melting point. Study of the effects of the addition of Co and V elements on ceramic particle size, interface binding, element distribution and the mechanical properties of (TiC_x_N_y_+TiB_2_)/Ni cermets prepared by in situ reaction of the Ni–Ti–B_4_C–BN system [32,33,34,35,36] and analysis of the differences between the influence of the two elements have important value and significance [37,38,39,40].

In this study, the Ni–Ti–B_4_C–BN system was selected as the research object. Hybrid TiC_x_N_y_+TiB_2_/Ni cermets were prepared by a combined combustion synthesis and hot-pressing method. The effects of Co and V contents on the ceramic particle size, interface binding, element distribution and mechanical properties of (TiC_x_N_y_+TiB_2_)/Ni cermets were investigated.

## 2. Experimental Procedures

**Figure 1:**
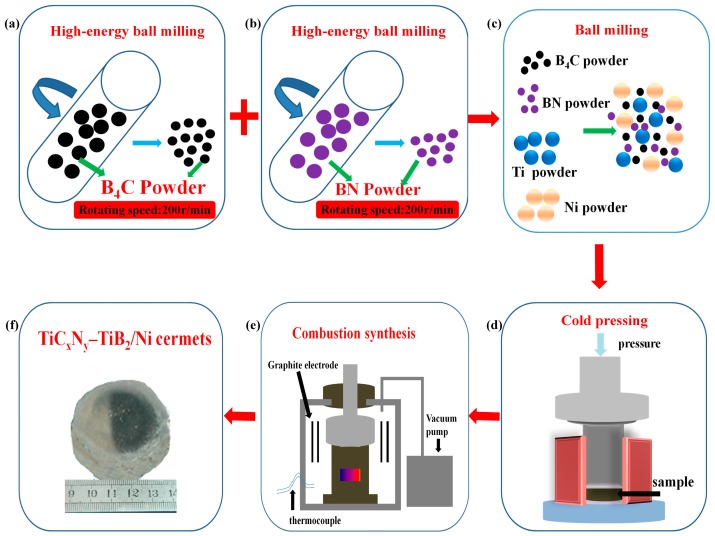
Schematic diagram of the preparation principle of the (TiC_x_N_y_–TiB_2_)/Ni cermets. (**a**) High-energy ball milling of B_4_C powder. (**b**) High-energy ball milling of BN powder. (**c**) Ball milling treatment of mixed powders. (**d**) Cold pressing mixed powders into a cylinder. (**e**) Preparation of micro-sized (TiC_x_N_y_–TiB_2_)/Ni cermets by hot press sintering of the compacts. (**f**) Sample of (TiC_x_N_y_–TiB_2_)/Ni cermets.

Figure 1 is a schematic diagram of the preparation principle of the TiC_x_N_y_–TiB_2_/Ni cermets. The raw materials used were commercial Ni powders (99.7 wt.% purity, ~25 μm in diameter, Beijing nonferrous metals research institute, Beijing, China), titanium powders (99.5 wt.% purity, ~58 μm in diameter, Beijing nonferrous metals research institute, Beijing, China), BN powders (99.3 wt.% purity, ~3 μm in diameter, Liaobin chemical company, Yingkou, Shandong, China), B_4_C powders (99.3 wt.% purity, ~3.5 μm in diameter, Zhengxing abrasive company, Dunhua, Jilin, China), Co powders (99.5 wt.% purity, ~47 μm in diameter, Beijing nonferrous metals research institute, Beijing, China) and V powders (99.7 wt.% purity, ~47 μm in diameter, Beijing nonferrous metals research institute, Beijing, China). As shown in Figure 1a,b, high-energy ball milling (Gosta, Siping, Jilin, China) was used to activate the boron carbide and boron nitride powders (Beijing nonferrous metals research institute, Beijing, China)at a rating speed of 200 r/min. The mole ratio of Ti:B_4_C:BN was fixed at 9:2:2, corresponding to the reaction in Equation (1).

(1) $${9\mathrm{Ti}+2B}_{4}C+2\mathrm{BN}\to 4\mathrm{Ti}\left(C_{0.5}N_{0.5}\right){+5\mathrm{TiB}}_{2}$$

Co and V powders at weight percentages of 2, 5 and 8 wt.% were added to the mixed powders ceramic particles at 70 wt.%, and the mixed powders were mixed with a stainless steel mixer (Gosta, Siping, Jilin, China) at a low speed (50 r/min) for 8 h, as shown in Figure 1c. The powders were pressed into a cylindrical shape on a cold press. The sample was then placed in a vacuum thermal explosion furnace (Gosta, Siping, Jilin, China), as shown in Figure 1e, and heated at a rate of 30 °K/min under vacuum. A sharp increase in barometric pressure indicated that the sample had reacted. The pressure was immediately applied to the sample for 60 s. Finally, the (TiC_x_N_y_–TiB_2_)/Ni cermets were successfully obtained by cooling to room temperature.

**Figure 2:**
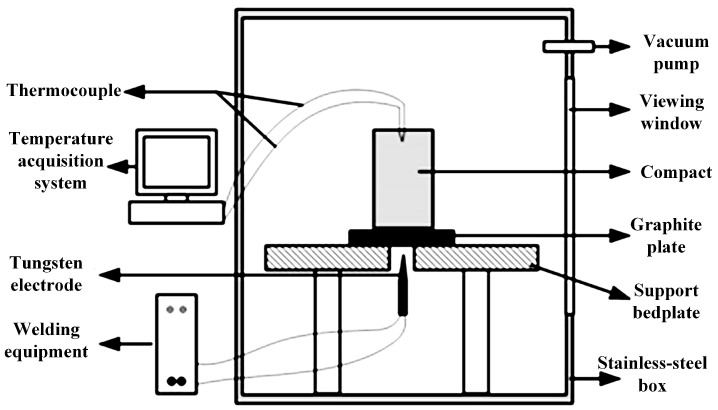
Self-propagating high-temperature synthesis (SHS) experimental device schematic.

The SHS experiment was carried out under a vacuum vessel (Qikun science, Hangzhou, zhejiang, China) in an Ar atmosphere. Figure 2 shows the schematic diagram of the device for the SHS experiment. In this experiment, arc heating was used, and the current of the arc welding was 85 A. The temperature of the sample was measured by W5–Re25 thermocouples (Kaitai, Shanghai, China). During the sample reaction, the data measured by the thermocouples were recorded and processed by a computer acquisition system (Hewlett-Packard development company, Changchun, Jilin, China).

The phase constituent of the sample was investigated by X-ray diffraction (XRD, Model D/Max 2500PC Rigaku, Tokyo, Japan) with Cu Kα radiation. The microstructure was investigated via scanning electron microscopy (SEM, Model Evo18 Carl Zeiss, Oberkochen, Germany). Density was measured by the drainage method according to the Archimedes principle. A servo-hydraulic materials testing system (MTS, MTS 810, Minneapolis, MN, USA) was applied to the compression test with a strain rate of 1 × 10^−4^ s^−1^ at room temperature. Cylindrical specimens with a diameter of 3 mm and a height of 6 mm were used for the compression tests. A Vickers hardness tester (Model 1600-5122VD, Laizhou, Shandong, China) was used to test the microhardness of the cermets. The standard Charpy impact test was conducted using a semiautomatic impact tester (JBW-300B, JNKH, Jinan, Shandong, China).

## 3. Results and Discussion

**Figure 3:**
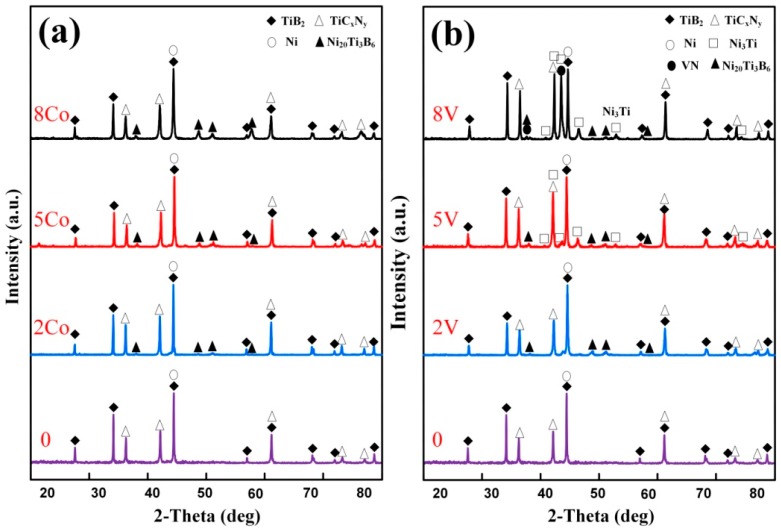
X-ray diffraction patterns for the (TiC_x_N_y_–TiB_2_)/Ni cermets. (**a**) (TiC_x_N_y_–TiB_2_)/Ni cermets with different Co contents (Co content from bottom to top is 0, 2, 5 and 8 wt.%, respectively). (**b**) (TiC_x_N_y_–TiB_2_)/Ni cermets with different V contents (V content from bottom to top is 0, 2, 5 and 8 wt.%, respectively).

Figure 3a shows XRD patterns for the (TiC_x_N_y_–TiB_2_)/Ni cermets with 0, 2, 5 and 8 wt.% Co addition. When Co was not added, the products contained only TiB_2_, TiC_x_N_y_ and Ni phases. After the addition of Co, only a small amount of Ni_20_Ti_3_B_6_ phase appeared, and the other phases did not change. Therefore, the addition of Co had no effect on the cermet reaction. Figure 3b shows the XRD patterns for the (TiC_x_N_y_–TiB_2_)/Ni cermets with 0, 2, 5 and 8 wt.% V addition. When V was not added, the products in the system were relatively pure, containing only TiB_2_, TiC_x_N_y_ and Ni phases. As the content of V increased from 2 to 5 wt.%, Ni_20_Ti_3_B_6_ and Ni_3_Ti phases appeared one after the other. When the V content continued to increase to 8 wt.%, in addition to Ni, TiB_2_, TiC_x_N_y_ and Ni_20_Ti_3_B_6_, a new phase, VN, appeared. This indicates that as the content of V increased, the reaction of Ni–V–Ti–B_4_C–BN system was not complete, and incomplete reactant and intermediate phases appeared successively. When the V content was increased to a certain extent, a large number of VN phases appeared in the system. Similar results were reported by Wang [41,42]. In the (TiC_x_N_y_–TiB_2_)/Ni composites without the addition of W, only the Ni, TiC_x_N_y_ and TiB_2_ phases are detected, while in the composites with the addition of W, a few Ni_20_Ti_3_B_6_ and Ni_3_Ti phases can be detected. It can be explained by the decline in the combustion temperature of the systems, which will be discussed below. Therefore, the addition of V, Co and W elements in the Ni–Ti–B_4_C–BN system causes incomplete reaction.

**Figure 4:**
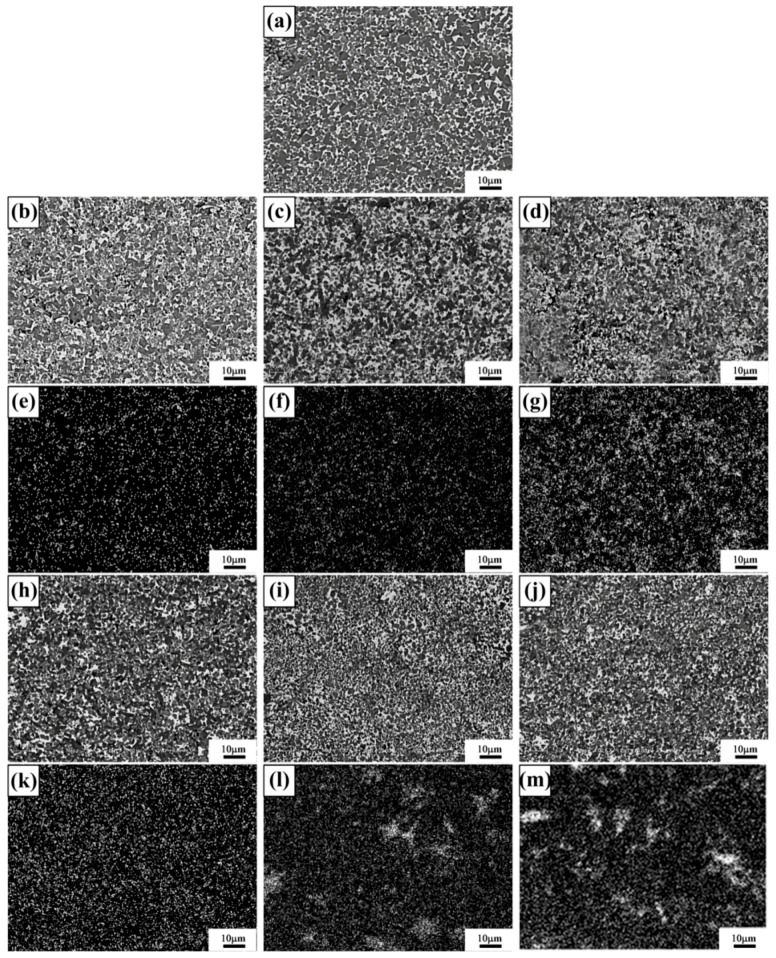
Microstructures of the (TiC_x_N_y_–TiB_2_)/Ni cermets with different Co and V contents. (**a**) SEM images of (TiC_x_N_y_–TiB_2_)/Ni cermets with 30 wt.% Ni. (**b**–**d**) and (**e**,**f**) SEM and EDS images of 70 wt.% (TiC_x_N_y_–TiB_2_)/Ni cermets with 2, 5 and 8 wt.% Co, respectively. (**h**–**j**) and (**k**–**m**) SEM and EDS images of 70 wt.% (TiC_x_N_y_–TiB_2_)/Ni cermets with 2, 5 and 8 wt.% V, respectively.

**Figure 5:**
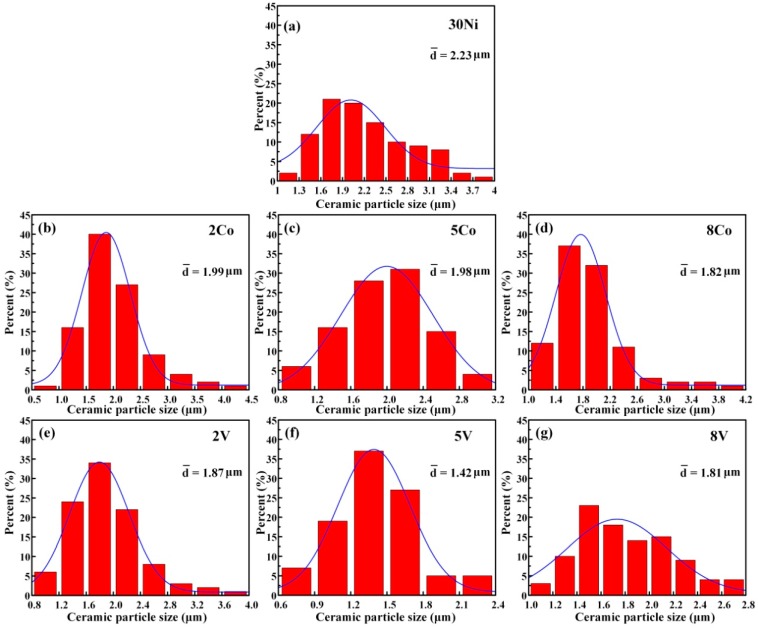
Size distribution diagrams of ceramic particles in the (TiC_x_N_y_–TiB_2_)/Ni cermets. (**a**) (TiC_x_N_y_–TiB_2_)/Ni cermets with 30 wt.% Ni. (**b**–**d**) 70 wt.% (TiC_x_N_y_–TiB_2_)/Ni cermets with 2, 5 and 8 wt.% Co, respectively. (**e**–**g**) 70 wt.% (TiC_x_N_y_–TiB_2_)/Ni cermets with 2, 5 and 8 wt.% V, respectively.

**Figure 6:**
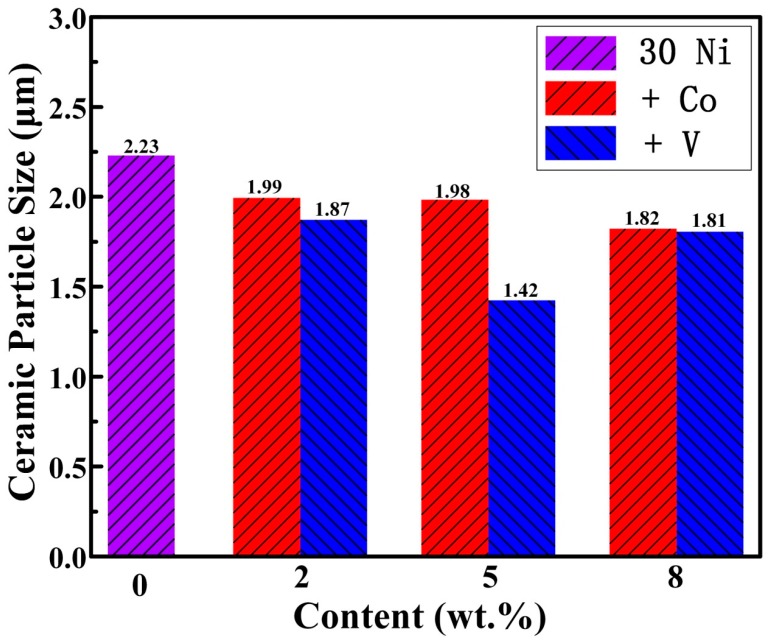
Average size of ceramic particles with different Co and V contents.

Figure 4 shows the microstructures of the (TiC_x_N_y_–TiB_2_)/Ni cermets with different Co and V contents. Figure 5 shows the size distribution diagrams of ceramic particles in the (TiC_x_N_y_–TiB_2_)/Ni cermets with different Co and V contents. As shown in Figure 4b–d, the ceramic particle size in the alloy did not change much, indicating that the Co content had little effect on the ceramic particle change. Figure 4e–g show energy-dispersive spectra (EDS) of (TiC_x_N_y_–TiB_2_)/Ni cermets with different Co contents. The white phase was a Co-rich phase. As the Co content increased, the enrichment zone of Co increased. Co did not participate in the reaction and acted only as a diluent, so it could only reduce the maximum combustion temperature of the reaction system to a smaller extent than the Group VIB alloying elements. As the Co content increased, the size of the ceramic particles was gradually reduced, as shown in Figure 5b–d and Figure 6.

Figure 4h–j shows the SEM images of the (TiC_x_N_y_–TiB_2_)/Ni cermets with different V contents. The ceramic particles in the (TiC_x_N_y_–TiB_2_)/(Ni+V) cermets were uniformly distributed. The size of the ceramic particles decreased first and then increased with the increase in V content, as shown in Figure 5e–g. When the V content was 5 wt.%, the ceramic particle size was at least 1.42 μm. With the addition of W and Al, and the sizes of the ceramic particles also decrease to less than 2 μm [42,43]. Figure 6 shows a statistical plot of the average size of ceramic particles with different amounts of Co and V cermets. The alloying element increased from 0 to 5wt.%, and the refinement effect of V on ceramic particles was more significant. When the V and Co contents were continuously increased to 8 wt.%, the ceramics in the cermets in which the Co and V elements were respectively added were similar in size. The effect of V addition in Ni–Ti–B_4_C–BN system on the size of ceramic particles is stronger than that of adding W, Al and Co [42,43].

**Figure 7:**
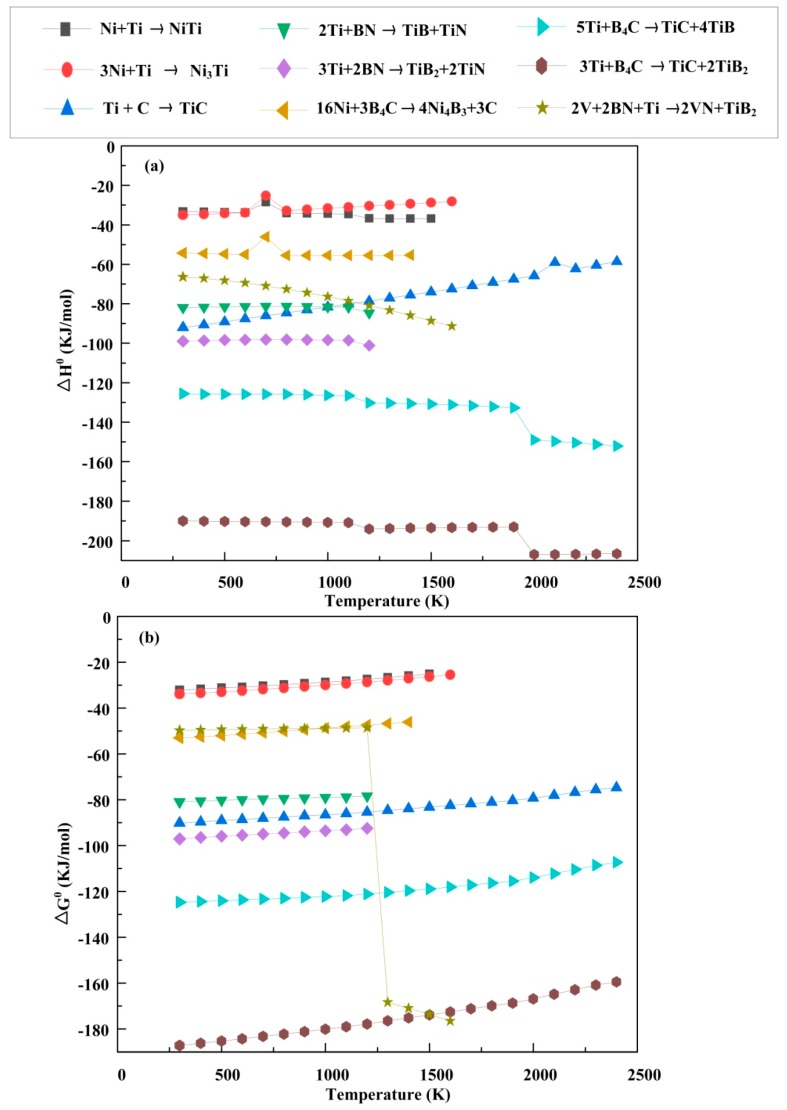
(**a**) ΔH^0^ and (**b**) ΔG^0^ of the latent reactions in Ni–(V/Co)–Ti–B_4_C–BN systems with the change of temperature (K).

Figure 7a,b shows the changes in reaction enthalpy (ΔH^0^) and standard Gibbs free energy (ΔG^0^) for the latent reactions in Ni–(V/Co)–Ti–B_4_C–BN systems with the change of temperature, respectively. Equations (2)–(10) are the reactions between each reactant or some intermediate phases. Moreover, certain proportions of TiC and TiN were used to represent the TiCN, overcome the relative complexity of the TiCN thermodynamic data and ignore the effect of TiC+TiN on TiCN.

Ni + Ti → NiTi(2)

3Ni + Ti → Ni_3_Ti (3)

Ti + C → TiC(4)

2Ti + BN → TiB + TiN(5)

3Ti + 2BN → TiB_2_ + 2TiN (6)

16Ni + 3B_4_C → 4Ni_4_B_3_ + 3C (7)

5Ti + B_4_C → TiC + 4TiB (8)

3Ti + B_4_C → TiC + 2TiB_2_ (9)

2V + 2BN + Ti → 2VN + TiB_2_ (10)

All of the reactions were thermodynamically feasible, with a negative Gibbs free energy exhibited in the calculated temperature range. Furthermore, the reaction enthalpy suggested that reactions (2)–(10) were all exothermal (ΔH^0^ < 0). Reaction (9) had the lowest reaction enthalpy and a far more negative standard Gibbs free energy, suggesting that TiC and TiB_2_ were the most stable products and that this reaction had the largest thermodynamics driving force contract with other reactions. TiN and TiB were less stable products, while NiTi, Ni_3_Ti and Ni_4_B_3_ were the most unstable intermediate products, as reactions (2), (3) and (7) showed relatively less heat release and a higher Gibbs free energy and ultimately transformed into more stable products. As Figure 7b shows, the Gibbs free energy of reaction (10) remained constant at first and dropped dramatically when the temperature exceeded 1200 °C, suggesting that VN was more stable under elevated temperatures.

**Figure 8:**
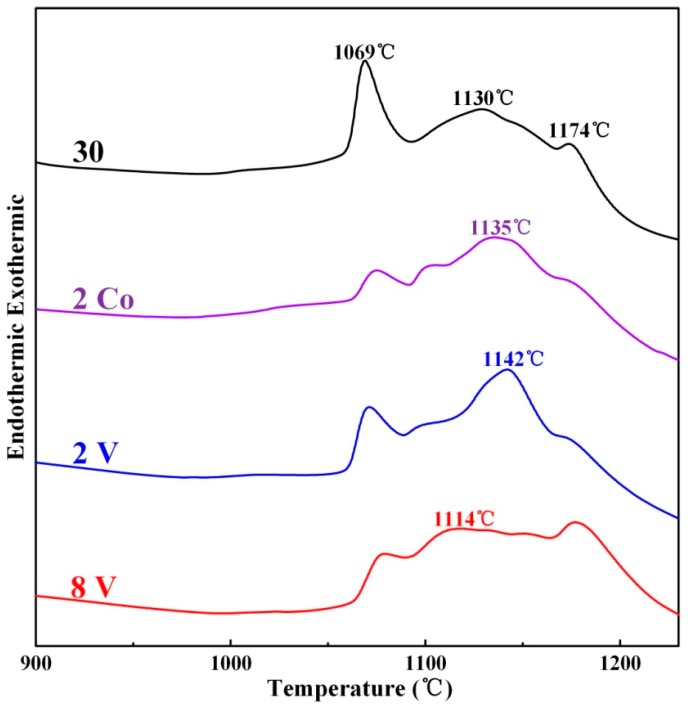
Differential thermal analysis (DTA) curves of different contents of Co/V in Ni–Ti–B_4_C–BN systems.

**Figure 9:**
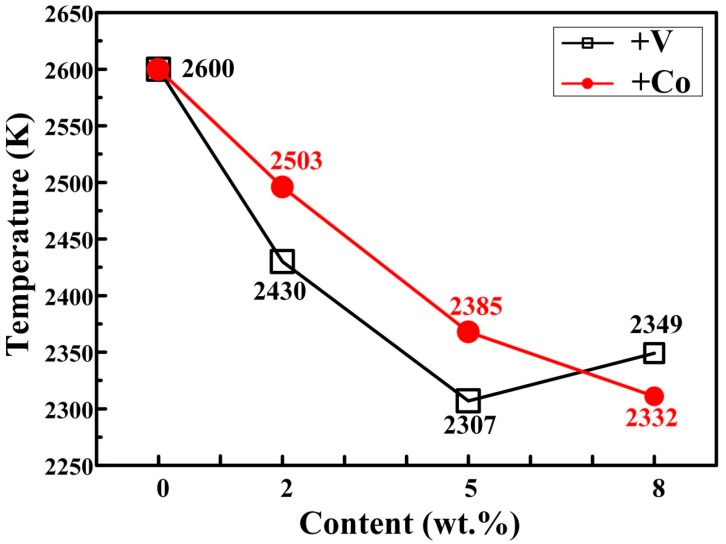
Combustion temperature curve of Ni–Ti–B_4_C–BN systems with different Co/V contents.

Figure 8 shows the DTA curves of different contents of Co/V in Ni–Ti–B_4_C–BN systems. Adding Co and V to the alloy had different effects on the reaction temperature of the system. Co played a role in dilution in the reaction system, so after the addition of Co, the reaction was delayed. With the addition of Co, the maximum combustion temperature continued to decrease, as shown in Figure 9. Although V participated in the reaction, a small added amount increased the maximum exothermic peak temperature. When the V content reached 8 wt.%, the amount of VN generated rose sharply, a large amount of heat was released and the reaction temperature of the system increased. Therefore, the reaction temperature of the system after the addition of V decreased first and then increased, as shown in Figure 9. The heat of fusion of V was larger than that of Co, so when the alloying element content was less than 5 wt.%, the reaction temperature of the alloy to which the V element was added decreased more than that of the Co element.

The highest combustion temperature is an important factor in the growth of ceramic particles, and a low reaction temperature leads to a size reduction of ceramic particles in composites. Figure 9 shows the combustion temperature curve of Ni–Ti–B_4_C–BN systems with different Co/V contents. The Co element did not participate in the SHS reaction and was a diluent; therefore, when the Co element was added, the combustion temperature continued to decrease. When the V content was no more than 5 wt.%, as the V content increased, the maximum combustion temperature decreased. When the content of V was less than 5 wt.%, the concentration of V was not sufficient to greatly promote the generation of VN. Therefore, V absorbed a large amount of heat during the reaction, resulting in a continuous decrease in the reaction temperature of the reaction system during the reaction. When the content of the added V continued to increase to 8 wt.%, V participated in the reaction, which was exothermic. This resulted in a higher temperature during the reaction than the maximum combustion temperature of the reaction in the system in which 5 wt.% of the alloying element V was added. Therefore, the average size of the ceramic particles in the cermets with 8 wt.% V content was larger than that with 5 wt.% V added. With the addition of W and Al, and the sizes of the ceramic particles continues to decrease due to the reduction of the maximum combustion temperature in the Wang’s works and our previous works [42,43].

**Figure 10:**
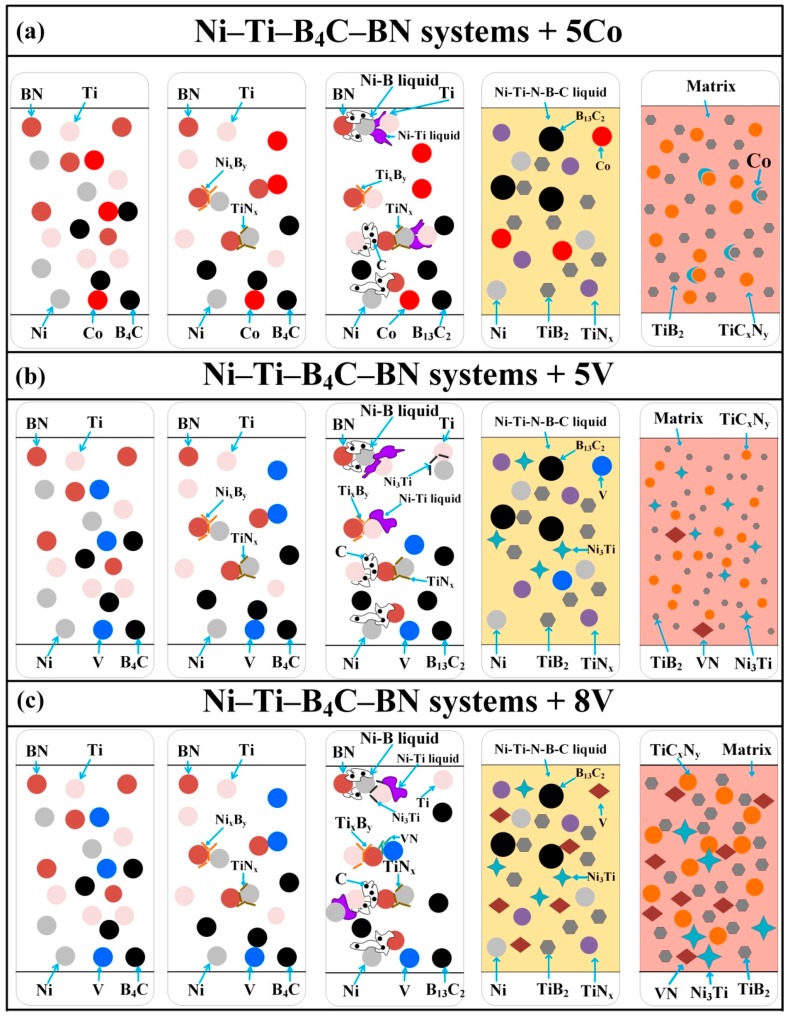
Reaction mechanism diagram of Ni–Ti–B_4_C–BN systems (**a**), (**b**) and (**c**) are the reaction mechanisms of Ni–Ti–B_4_C–BN systems with 5 wt.% Co, 5 wt.% V and 8 wt.% V, respectively.

By analyzing the XRD, SEM and EDS of the Ni–Ti–B_4_C–BN–Co/V system, the influence of V and Co on the reaction mechanism of Ni–Ti–B_4_C–BN system could be established, as shown in Figure 10. In the Ni–Ti–B_4_C–BN system, the solid-solid reactions between Ni and B_4_C and between Ti and BN occurred first, respectively forming Ni_2_B, Ni_4_B_3_ and C and TiN_x_ and B. Ni then reacted with TiN_x_ to form NiTi and Ni_3_Ti. As the temperature increased, the Ni–B and Ni–Ti liquid phases formed between Ni_2_B and Ni_4_B_3_ and between NiTi and Ni_3_Ti, respectively. Once the liquid phases were formed, they rapidly spread on the surface of the unreacted particles, greatly promoting the dissolution of B, N and C atoms in the liquid phase and thereby forming a Ni–Ti–B–N–C liquid phase. When the concentrations of [Ti], [B], [C] and [N] in the liquid phase of Ni–Ti–B–N–C satisfied the thermodynamic conditions for the formation of TiB_2_ and TiC_x_N_y_, the reaction formed TiB_2_ and TiC_x_N_y_ and precipitated from the liquid phase. As shown in Figure 10a, Co did not participate in the reaction, but had an inhibitory effect on the synthesis process by diluting the reactant. Finally, a fraction of the Co atoms entered the Ni to form a solid solution in the matrix, while the remaining Co atoms were segregated around the interface between the ceramic particles. In the Ni–Ti–B_4_C–BN–V systems, when the V content was 5 wt.%, as shown in Figure 10b, the concentration of VN was not greatly promoted due to insufficient concentration; V acted only to refine the particles, and the ceramic particles were the smallest at this time. When the V content reached 8 wt.%, the increase in V concentration greatly promoted the generation of VN, the reaction temperature of the system increased and the size of the ceramic particles increased.

**Figure 11:**
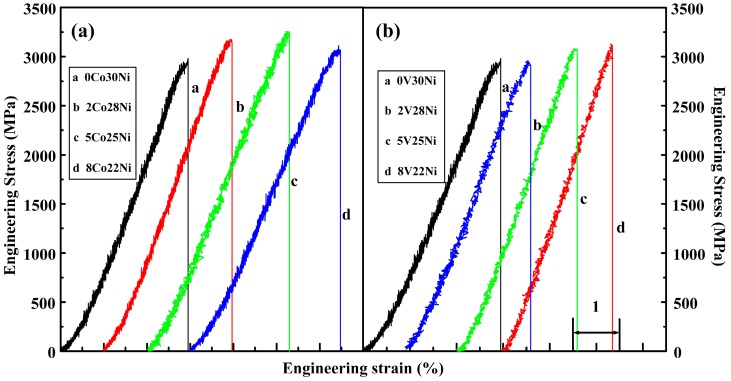
Compression engineering stress-strain curves of the (TiC_x_N_y_–TiB_2_)/Ni cermets. (**a**) (TiC_x_N_y_–TiB_2_)/Ni cermets with different Ni and Co contents. (**b**) (TiC_x_N_y_–TiB_2_)/Ni cermets with different Ni and V contents.

Figure 11 shows the compressive engineering stress-strain curves for (TiC_x_N_y_–TiB_2_)/(Ni+Co/V) cermets with different Co and V contents. The addition of Co and V elements resulted in a different degree of improvement in the mechanical properties of the cermets. Table 1 lists the hardness and mechanical properties of (TiC_x_N_y_–TiB_2_)/Ni cermets with different Co and V contents. As the Co content increased, the relative density of the cermets increased first and then decreased. The addition of Co was beneficial to the wetting between ceramic particles and nickel, thus contributing to the densification of the cermets. With the Co content increased, the reaction temperature of the system decreased; the reduction of the reaction temperature was not conducive to densification. Therefore, when the Co content was 8 wt.%, the relative density was reduced. Figure 11a shows the compressive engineering stress-strain curves for (TiC_x_N_y_–TiB_2_)/(Ni+Co) cermets with different Co contents. With the increase in Co content from 0 to 5 wt.%, the ultimate compressive strength ($\sigma_{\mathrm{UGS}}$) and fracture strain ($\varepsilon_{f}$) increased from 2.94 to 3.25 GPa and 2.9% to 3.25%, respectively. Then, as the Co content continued to increase, both $\sigma_{\mathrm{UGS}}$ and $\varepsilon_{f}$ decreased. When the Co content was 5 wt.%, the hardness of the cermets reached the highest value of 1967 Hv. Impact toughness is an important property for brittle cermets [44,45]. The impact properties of (TiC_x_N_y_–TiB_2_)/(Ni+Co/V) cermets with different Co and V contents are shown in Table 1. As the Co content increased, the impact toughness of the cermets increased from 2.94 and 6.05 to 7.52 GPa. The addition of V reduced the impact properties of (TiC_x_N_y_–TiB_2_)/(Ni+V) cermets. The addition of Co strengthens the impact toughness of the (TiC_x_N_y_–TiB_2_)/Ni cermets, while the addition of V deteriorates it. Obviously, the addition of a small amount of Co promoted the wetting of ceramic particles and Ni was beneficial to the densification of the cermets, strengthened the interfacial bonding between the ceramic particles and Ni, improved the strength and hardness of the cermets, and reduced the density, strength and hardness when the amount of addition was too much.

As the V content increased, the relative density of the (TiC_x_N_y_–TiB_2_)/(Ni+V) cermets increased continuously. Unlike the addition of the Co element, the relative density of the cermets with V added increased as the V content increased; when the V element reached a certain content, the relative density did not change. The hardness of the cermets increased as the V content increased. Figure 11b shows the compressive engineering stress–strain curves for (TiC_x_N_y_–TiB_2_)/(Ni+V) cermets with different V contents. As the V content increased, the $\sigma_{\mathrm{UGS}}$ of the cermets increased, while the $\varepsilon_{f}$ decreased. When the content of V was 8 wt.%, the cermets had the highest compressive strength of 3.11 GPa and the minimum $\varepsilon_{f}$ was 2.3%. The hardness of the cermets reached the maximum level. When the V content in the cermets reached 8 wt.%, there was a large amount of VN formation in the alloy system, and the ceramic content in the cermets increased, which led to a large increase in the strength and hardness of the (TiC_x_N_y_–TiB_2_)/(Ni+Co) cermets.

**Figure 12:**
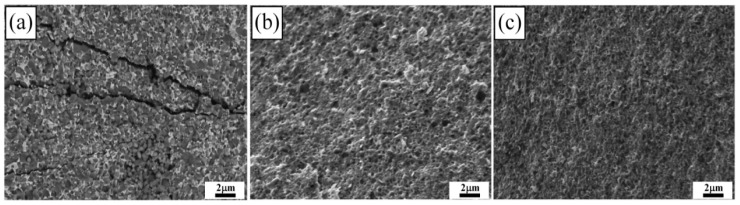
SEM images of the compression fractured surfaces for (TiC_x_N_y_–TiB_2_)/Ni cermets. (**a**) (TiC_x_N_y_–TiB_2_)/Ni cermets with no alloy element. (**b**) (TiC_x_N_y_–TiB_2_)/Ni cermets with 5 wt.% Co. (**c**) (TiC_x_N_y_–TiB_2_)/Ni cermets with 5 wt.% V.

Figure 12 shows SEM images of the compression fractured surfaces for (TiC_x_N_y_–TiB_2_)/Ni cermets with 5 wt.% Co and V added. As indicated, in the (TiC_x_N_y_–TiB_2_)/Ni cermets without the addition of Co and V, most of the cracks propagated along the ceramics–Ni interface, while in the cermets with the addition of Co and V, there were no obvious cracks on the surface of the fracture. This indicates that the addition of Co and V in the cermets increased the bonding strength between the Ni and ceramic particles, thereby increasing the compressive strength and toughness of the cermets.

**Figure 13:**
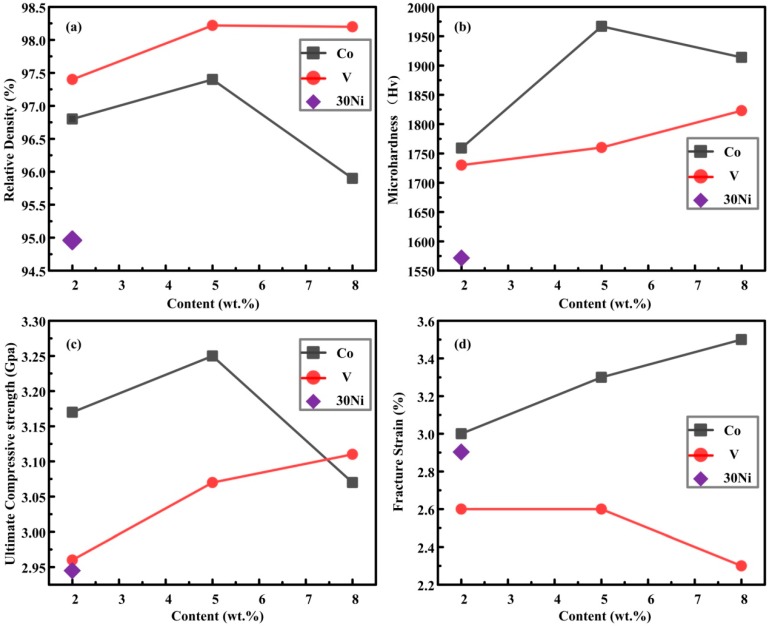
Variations of (**a**) relative density, (**b**) microhardness (Hv), (**c**) ultimate compressive strength $\sigma_{\mathrm{UGS}}$ and (**d**) fracture strain $\varepsilon_{f}$ of (TiC_x_N_y_–TiB_2_)/Ni cermets with different Co/V contents.

Figure 13 shows variations of the (a) relative density, (b) microhardness, (c) ultimate compressive strength and (d) fracture strain of (TiC_x_N_y_–TiB_2_)/Ni cermets with different Co/V contents. The addition of Co was more effective than the addition of V for the ultimate compressive strength, the impact toughness and microhardness of the (TiC_x_N_y_–TiB_2_)/Ni cermets. The addition of the V element mainly refined the ceramic particles in the cermets; therefore, as the V content increased, the microhardness and ultimate compressive strength of the cermets increased. The addition of Co and V reduced the reaction temperature of the system, but the heat of fusion of V was 21.5 kJ/mol^−1^ higher than that of Co 16.06 kJ/mol^−1^, so the reaction temperature of V was lower than that of Co. As the V content increased, the number of ceramic particles increased, which reduced the content of Ni in the cermets and reduced the shaping of the cermets. Hence, as the V content increased, the fracture strain of the cermets decreased. The following factors affected the influence of the addition of the Co element on the cermets. First, Co could form an infinite solid solution with Ni in the matrix. The more Co atoms dissolved in the Ni, the stronger the solid solution became. Second, as the Co content increased, the size of the cermets’ ceramic particles decreased slightly. Therefore, when the Co content increased from 0 to 5 wt.%, the hardness and compressive strength of the cermets also gradually increased. Ultimately, the addition of Co promoted the wetting of ceramic particles and Ni and strengthened the interfacial bonding between the ceramic particles and Ni. However, when Co was continuously added to the Ni–Co–Ti–B_4_C–BN systems to 8 wt.%, the density of the cermets decreased, resulting in a decrease in performance of the cermets.

## 4. Conclusions

Microstructure and mechanical properties of (TiCxNy–TiB2)/Ni cermets with different Co and V contents (2, 5 and 8 wt.%) fabricated by combustion synthesis and hot press consolidation were studied. The addition of Co and V elements refined the ceramic particles to different degrees. Co did not participate in the reaction, but had an inhibitory effect on the synthesis process by diluting the reactant. When the V content was 5 wt.%, V acted only to refine the particles, and the ceramic particles were the smallest at this time. When the V content reached 8 wt.%, the increase in V concentration greatly promoted the generation of VN, the reaction temperature of the system increased and the size of the ceramic particles increased. The heat of fusion of V was higher than that of Co, so the decrease in the temperature and the refinement effect on the ceramic particles of the V element were more significant. The cermets with 5 wt.% Co possessed the best comprehensive properties: the highest hardness (1967 Hv), a superior compression strength (3.25 GPa) and a higher fracture strain (3.3%), which are 406 Hv, 0.31GPa and 0.4% higher than those of the unalloyed cermets. Correspondingly, when the V content was 8 wt.%, those three values reached 1823 Hv, 3.11 GPa and 2.3%, respectively. The addition of Co acted as a fine-grained strengthening and solid solution strengthening, and Co promoted the wetting of ceramic particles and Ni and strengthened the interfacial bonding between the ceramic particles and Ni. Furthermore, as the V content increased to 8 wt.%, the number of ceramic particles increased, which reduced the Ni content and thereby the shaping of the cermets. Therefore, the addition of Co is more significant than the addition of V to the mechanical properties of the cermets.

## Figures and Tables

**Table 1:** Room-temperature compression properties, impact toughness, microhardness and density of (TiC_x_N_y_–TiB_2_)/Ni cermets with different Co and V contents.

Sample	Density			Hv	$\sigma_{\mathrm{UGS}}\;(\mathrm{GPa})$	$\varepsilon_{f}\;(\%)$	*K*c (MPa·m^1/2^)
	Theoretical Density (g/cm^3^)	Measured Density (g/cm^3^)	Relative Density (%)				
30 wt.% Ni	5.52	5.24	94.8	1561 ± 31	$2.94_{-0.3}^{+0.5}$	$2.9_{-0.4}^{+0.6}$	6.05 ± 0.25
28 wt.% Ni + 2Co	5.52	5.35	96.8	1759 ± 52	$3.17_{-0.4}^{+0.8}$	$3.0_{-0.5}^{+1.3}$	6.56 ± 0.18
25 wt.% Ni + 5Co	5.52	5.39	97.4	1967 ± 40	$3.25_{-0.3}^{+0.6}$	$3.3_{-0.3}^{+0.7}$	6.97 ± 0.31
22 wt.% Ni + 8Co	5.52	5.29	95.9	1914 ± 51	$3.07_{-0.5}^{+0.4}$	$3.5_{-0.6}^{+0.9}$	7.52 ± 0.33
28 wt.% Ni + 2V	5.48	5.34	97.4	1730 ± 25	$2.96_{-0.9}^{+0.7}$	$2.6_{-0.7}^{+0.4}$	5.76 ± 0.25
25 wt.% Ni + 5V	5.43	5.34	98.2	1760 ± 63	$3.07_{-0.6}^{+0.3}$	$2.6_{-0.6}^{+0.8}$	5.93 ± 0.16
22 wt.% Ni + 8V	5.38	5.28	98.2	1823 ± 45	$3.11_{-0.9}^{+1.2}$	$2.3_{-0.9}^{+0.5}$	5.65 ± 0.17
